# Effect of Endo-Z bur or Bladesonic ultrasonic tip on the adaptation of filling material. A micro-CT study

**DOI:** 10.1590/0103-6440202305474

**Published:** 2023-12-22

**Authors:** Pedro Henrique Fiorin de Souza, Airton Oliveira, Jáder Camilo Pinto, Karina Ines Medina Carita Tavares, Juliane Maria Guerreiro-Tanomaru, Mário Tanomaru-Filho

**Affiliations:** 1 Department of Restorative Dentistry, School of Dentistry, UNESP - Universidade Estadual Paulista, Araraquara, SP, Brazil; 2 Departament of Dentistry - Centro Universitário Presidente Antônio Carlos - UNIPAC, Barbacena, MG, Brazil and Department of Dentistry - Centro Universitário Presidente Tancredo de Almeida Neves - UNIPTAN, São João del Rei, MG, Brazil

**Keywords:** apicoectomy, endodontics, ultrasound, x-ray microtomography

## Abstract

This study aimed to evaluate the effect of apicoectomy performed with a bur or an ultrasonic tip on the adaptation of the filling material to root canal dentin. Twenty human mandibular incisors were selected and prepared with a ProDesign Logic system up to diameter 40/.05. The root canals were filled with Bio-C Sealer (Angelus, PR, Brazil) using the single cone technique and were stored at 37ºC and 95% relative humidity for 7 days. After this period, the apicoectomy of the 3 millimeters of the root apex was performed using Endo-Z bur (Dentsply Sirona) or Bladesonic ultrasonic tip (Helse Ultrasonic, Santa Rosa de Viterbo, Brazil) (n=10). The specimens were scanned by micro-computed tomography (micro-CT; SkyScan 1176) with a voxel size of 8.74 µm after obturation and after apicoectomy. The percentage of voids at the interface between the filling material and the root canal wall was evaluated in the apical 4 mm of the root after apicoectomy, besides the time cutting to both devices. The data obtained were submitted to paired and unpaired t-tests (α = 0.05). Endo-Z and Bladesonic increased the percentage of voids between the filling material and the dentin after apicectomy (p<0.05), and they were similar (p>0.05). Endo-Z has a shorter time to perform apicoectomy compared to Bladesonic (p<0.05). Apicectomies with Endo-Z or Bladesonic could harm the material/dentin interface, while the Endo-Z bur was faster than the Bladesonic ultrasonic tip.

## Resumo

O objetivo deste estudo foi avaliar o efeito da apicectomia realizada com broca ou inserto ultrassônico na adaptação do material obturador á dentina do canal radicular. Vinte incisivos inferiores humanos extraídos foram selecionados e preparados com o sistema ProDesign Logic até o diâmetro 40/.05. Os canais radiculares foram obturados com Bio-C Sealer (Angelus, PR, Brasil) por meio da técnica de cone único e foram armazenados em estufa a 37ºC e umidade relativa de 95% por 7 dias. Após esse período, foi realizada apicectomia dos 3 milímetros apicais utilizando broca Endo-Z (Maillefer - Dentsply) ou inserto ultrassônico BladeSonic (Helse Ultrasonic, Santa Rosa de Viterbo, Brasil) (n=10). Os espécimes foram escaneados por meio de microtomografia computadorizada (micro-CT; SkyScan 1176) com tamanho de voxel de 8,74 µm após obturação e após apicectomia. Please, correct this sentece to O percentual de falhas na interface entre o material obturador e a parede dentinaria foi avaliado nos 4 mm apicais da raiz apos apicectomia, alem do tempo de corte para ambos dispositivos. Os dados obtidos foram submetidos aos testes t pareado e não pareado (α = 0,05). Endo-Z e Bladesonic aumentaram o percentual de vazios entre o material obturador e a parede da dentina após apicectomia (p<0.05), sendo semelhantes entre si (p>0.05). Endo-Z demonstrou menor tempo para realizar apicectomia em comparação com Bladesonic (p<0.05). Apicectomias com Endo-Z ou Bladesonic podem prejudicar a interface material obturador/dentina, sendo a broca Endo-Z mais rápida que a ponta ultrassônica Bladesonic.

## Introduction

The failure of endodontic treatment is related to the persistence of the infection ^1^, voids in the filling material, the persistence of intra and extra-radicular biofilm ^2^, and the presence of periapical cysts ^3^. Endodontic retreatment or periradicular surgery is indicated in cases of failure and should provide favorable conditions for the repair of periapical tissues ^4^. However, endodontic retreatment has a lower success rate when compared to primary endodontic treatment, approximately 60-70 % ^5^. Furthermore, a lower retreatment success rate may be related to greater wear on the tooth structure ^6^.

Endodontic microsurgery has a high success rate between 89-100% ^7^. Apicectomy consists of removing the root apical portion and should provide a smooth and regular root surface, without impairing the adaptation of the filling material ^8^ ^,^ ^9^. Failures in the material/dentin interface may allow microleakage, negatively impacting the endodontic prognosis ^8^. According to Kim et al. ^1^, a section of 3 mm from the apical root portion provides a reduction of 93-98% of the ramifications of the Root Canal System. ^8^ ^,^ ^9^.

Apicoectomy can be performed using burs, lasers, and ultrasonic tips ^10^. The use of burs can provide apicectomy with the proper apical surface ^11^, however, it can affect the adaptation of the filling material ^10^. Therefore, the incorporation of new ultrasonic tips in the arsenal of endodontic microsurgery may represent important alternatives ^1^ ^,^ ^10^ ^,^ ^12^. Besides, ultrasonic tips favor the preparation in regions of difficult access ^10^.

A new ultrasonic tip Bladesonic (Helse Ultrasonic, Santa Rosa de Viterbo, Brazil) has been introduced in the market to perform apicoectomy and osteotomy. This tip is made of stainless steel with a 2.20 mm active tip of length and 0.30 mm thickness. However, there is no data considering the performance of Bladesonic in apicoectomies. Thus, it seems appropriate to test the Bladesonic ultrasonic tip performance, in comparison with the conventional technique using the Endo-Z bur.

Since apicoectomy is a step in periradicular surgery, with a possible effect on the adaptation of the filling material to the dentin wall, the present study aims to evaluate, by micro-CT, the effect of apicoectomy using Endo-Z bur or Bladesonic ultrasonic tip on the material/dentin interface. The null hypothesis tested was that both devices would not interfere with the adaptation of the filling material to the dentin walls after apicoectomy.

## Material and Methods

Twenty extracted, single canals, straight and circular mandibular incisors were selected and used after approval by the Ethics Committee for Research (Protocol CAAE No. 29568720.2.0000.5416). A digital radiography system (Kodak RVG 6100 Digital Radiography System, Marne-la-Vallée, France) was used to confirm the inclusion criteria and to perform a homogeneous distribution of the samples. The specimens were immersed in distilled water for 48 hours, and then a conventional access of the root canals was performed using high-speed diamond burs (n° 2, KG Sorensen, São Paulo, Brazil). The root canals were explored using a 10 K-file (Dentsply Sirona, Ballaigues, Switzerland) and the working length (WL) was established 1 mm short of the apical foramen, confirmed by digital radiographs. The roots were molded in condensation silicone (Oranwash, Zhermack SpA, Badia Polesine, Italy) to simulate the periodontal ligament.

The teeth were prepared using ProDesign Logic (PDL) 25/.04, 25/.06, and 40/.05 at a speed of 950 RPM and torque of 4 Ncm, in a rotary motion. All the specimens were irrigated with 1% sodium hypochlorite (NaOCl) (Ciclo Farma, Serrana, SP, Brazil) after the use of each instrument (2.5 mL), using an Ultradent syringe (South Jordan, UT, USA) with Navitip 30-G needle (Ultradent Products) at 2 mm short of the WL, with in-and-out motion, and continuous aspiration after each instrument. The final irrigation was performed with 5 mL of 2.5% NaOCl, and 2.5 mL of 17% EDTA (Biodinâmica, Ibiporã, PR, Brazil) under agitation for 3 minutes with a 40 K-file and 5mL physiological solution.

### Obturation of specimens and micro-CT scan

After preparation, the root canals were obturated by the single cone technique, using 40/.05 cones (Tanariman, Manacapuru, AM, Brasil) and Bio-C Sealer (Angelus, Londrina, PR, Brazil), which was inserted into the canal at 4 mm of the WL using a syringe applicator. After introducing the sealer into the root canal, the gutta-percha cone wrapped in endodontic sealer was taken into the canal. Next, the excesses of gutta-percha were cut at the cervical level with a heat plugger (Golgran, São Caetano do Sul, SP, Brazil). Buccolingual and mesiodistal radiographs were taken to assess the quality of the obturation in all specimens. The access cavities were sealed with Coltosol (Vigodent, Rio de Janeiro, Brazil) and the roots were stored in a stove at 37 °C and 95% humidity for 7 days to allow the final setting of the sealer. After this period, the teeth were randomly divided into two groups (n=10) and initial micro-CT scanning was performed (SkyScan 1176, Bruker, Kontich, Belgium) using the following parameters: 80 kV X-ray tube voltages, 300 uA anode current, rotation step 0.5, rotation angle 180°, frame averaging 4, Cu + Al filter, and isotropic voxel size of 8.74 µm.

### Apicoectomy

Root section was performed approximately 3 mm from the apex by an endodontic specialist with extensive experience in periradicular surgery using a dental operative microscope at 13x magnification. For the first group, apicoectomy was performed using the Endo-Z bur (Dentsply Sirona, York, Pennsylvania, USA) coupled to a low-speed motor (Micromotor N270 and Counter-angle; Dabi-Atlante, Ribeirão Preto, SP, Brazil) under constant irrigation. After creating a groove on the mesial surface, back-and-forth movements in the buccal lingual direction were performed until the root was completely sectioned. The direction of the apicoectomy cut was made in the same direction of the rotation.

**Figure 1:**
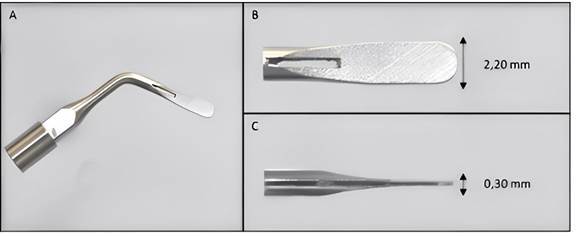
Demonstrative photographic images of the Bladesonic ultrasonic tip (Helse Ultrasonic, Santa Rosa de Viterbo, Brazil). General view of the insert (A), side view of the active part (B) and front view of the active part (C).

For the second group, a Bladesonic ultrasonic tip (Helse Ultrasonic, Santa Rosa de Viterbo, Brazil) (Figure 1) was used, coupled to the ultrasonic device Acteon (Indaiatuba, SP, Brazil) at a frequency of 50 Hz and power of 70% power under constant irrigation with distilled water, following the manufacturer's guidelines. A Bladesonic tip was used creating a groove on the buccal surface extending mesial and then a smooth back-and-forth movement was performed in the buccal lingual direction with light pressure and continuous contact with the mesial surface towards the distal surface. For finishing, circular movements were applied along the apical surface. For both experimental groups, the specimens were positioned in a delineator device to ensure stability during apicoectomy. The apical portion was cut at a 90º angle in relation to the long axis of the root, allowing standardization for both tested methods, as described by Berbert et al. ^10^.

### Final micro-CT scan and image processing

After apicoectomy, the specimens were scanned again following the same parameters as the initial scan. Specific software was used for reconstruction (NRecon v.1.6.3; SkyScan, Kontich, Belgium), 3D registration (Data Viewer v.1.5.1; Sky Scan) and quantitative image analysis (CTAn v.1.15.4.0; SkyScan). Differences in the percentages of voids between the surface of the root canal walls and the filling material before and after apicoectomy were measured from the method described by Gandolfi et al. ^14^. The 3D distribution of the interface of voids in a predefined volume of interest (VOI) was calculated at the last 4 mm of the root after apicoectomy. After defining the bottom and top, the adaptive thresholding was employed to recognize each object of interest. Each 3D-VOI was defined as the volume interface, embracing part of the dentin and part of the filling material by applying a ¨*task lists¨* with arithmetical and logical operations between the superimposed sections. Thus, voids with sizes starting at 8.74 μm inside the VOI were detected. Representative images of voids were created using CTAn software (v.1.15.4.0; SkyScan).

### Statistical analysis

The data were submitted to the Shapiro-Wilk test and presented as a normal distribution. A paired *t-test* was used to compare the percentage of voids between the root canal wall and the filling material before and after apicoectomy. An unpaired *t-test* was used to compare the increase in the percentage of voids and cutting time between the groups. The level of significance was 5% for all analyses.

## Results

**Figure 2:**
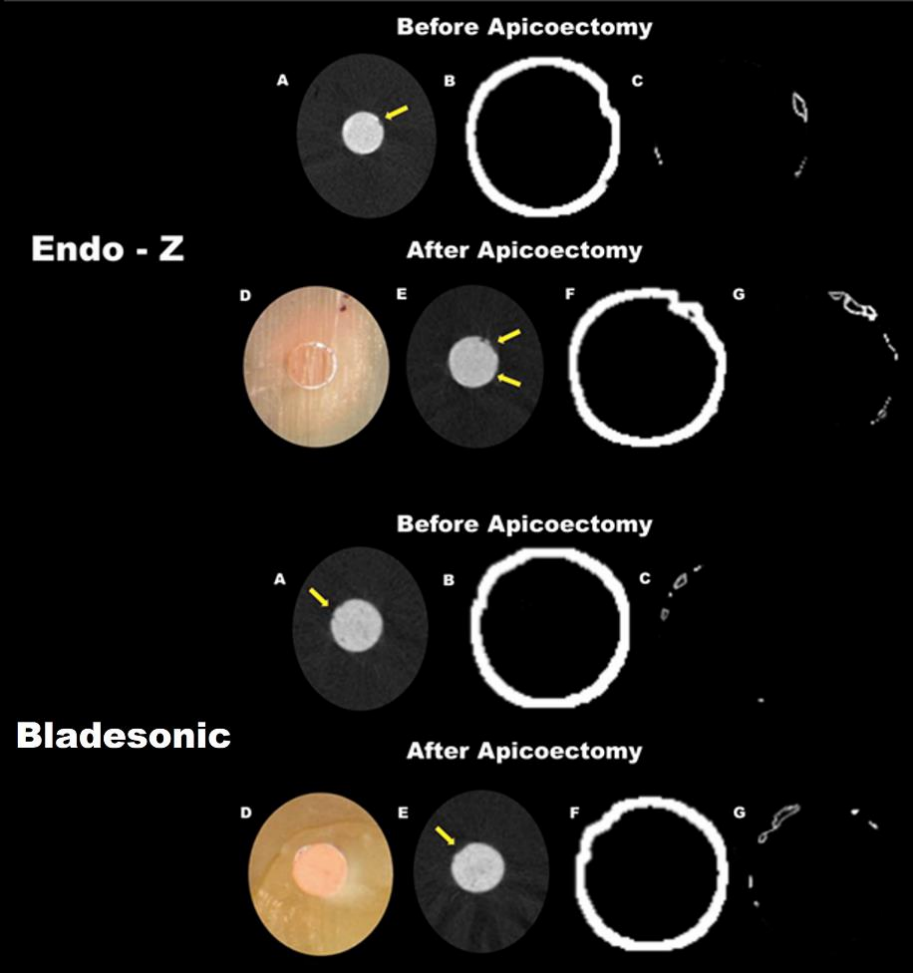
Representative images using CTAn software before and after apicoectomy using different devices. Root canals obturated with Bio-C Sealer (A and E), the yellow arrows point to the voids at the material/dentin interface; Images after apicoectomy using Endo-Z bur or Bladesonic ultrasonic tip, respectively (D); Volume interface: material/dentin (B and F); Presence of voids at the material/dentin interface (C and G).

Endo-Z and Bladesonic increased the percentage of voids between the root canal wall and the filling material after apicoectomy (p<0.05), with no significant difference between the groups (p>0.05). Regarding cutting time, Endo-Z had less time to perform apicoectomy compared to Bladesonic (p<0.05) (Table 1). The percentage of voids at the material/dentin interface for both devices before and after apicoectomy is shown in Figure 2.

**Table 1:** Percentage of voids between root canal wall and filling material, increase the percentage of voids after the apicoectomy and cutting time (mean and standard deviation).

	Before Apicoectomy	After Apicoectomy	Increase the percentage of voids (%)	Cutting time (min)
Endo-Z	7.22 ± 2.94^b^	12.81 ± 0.87^a^	69.55 ± 27.71^A^	1.25 ± 0.34^B^
Bladesonic	3.26 ± 1.72^b^	5.26 ± 2.12^a^	67.41 ± 27.16^A^	5.07 ± 1.12^A^

Different superscript lowercase letters in the same line indicate statistical differences between the same group before and after apicoectomy (p<0.05). Different superscript uppercase letters in the same column indicate statistical differences between the groups (p<0.05).

## Discussion

The adaptation of the filling material to the dentin wall can be affected by cutting the root promoting the formation of voids after the cutting procedure ^10^. These voids may allow infiltration by tissue fluids ^10^. In the present study, the null hypothesis was rejected, since Bladesonic and Endo-Z significantly increased the percentage of voids after apicoectomy, achieving a 69.55% increase of voids for Endo-Z and 67.41% for Bladesonic, being similar to each other. In agreement, the literature correlated apicoectomies with a bur in high-rotation with the formation of voids at the material/dentin interface (8, 9).

The direction of the bur during apicoectomy was indicated as an important factor for the incidence of displacement of the filling material ^8^. Burs used in the opposite direction to the cutting guidance cause greater displacement of the filling ^8^. However, in agreement with previous studies (8, 15) our results showed an increase in the percentage of voids, even using the direction indicated as favorable for Endo-Z bur, in the direction of the cutting guidance. The heat produced by burs at high-rotation ^10^ may impact the quality of the material/dentin interface ^15^. Analysis by scanning electron microscopy (SEM) showed greater displacement of the filling material by high-rotation burs compared to low-rotation burs ^15^.

Ultrasonic vibration during root cutting provides smooth and regular walls ^16^. However, the power used in the ultrasonic device increases the vibration amplitude of the ultrasonic tip ^17^, which may cause greater displacement of the filling material. In this study, following the manufacturer's recommendations, Bladesonic was activated at higher power (70%), resulting in a great percentage of increased voids at the material/dentin interface, similar to the Endo-Z. Adaptation of filling material to root dentin was similar after apicectomy using Zekrya bur (Dentsply/Maillefer) or CDV 9.5107-8 ultrasonic tip (CDV-Vale, São José dos Campos, Brazil) ^10^. This is the first study that evaluated the performance of the Bladesonic ultrasonic tip, providing a three-dimensional analysis of the interface between the filling material and dentin after apicoectomy.

The time used for apicectomy is a relevant clinical parameter ^10^. The shorter cutting time of the root apex can be related to less surgical trauma, greater comfort for the patient, and a more uniform and continuous section of the root surface ^11^. Our results showed a longer time for apicoectomy with Bladesonic compared to Endo-Z bur. Similar results were obtained in a previous study ^10^. However, considering the methodology employed in the present study, using extracted human teeth, not all the difficulties found in a clinical scenario during periradicular surgery were considered. Different angles, shapes, and sizes of ultrasonic tips may optimize the capacity of cutting during apicoectomy of teeth with difficult access ^10^.

The obturation technique and the endodontic sealer can influence the adaptation of the filling material to the root canal ^18^. The single cone technique was used in the present investigation because it allows greater standardization of filling material and root canal filling technique ^19^, and is less dependent on the operator ^20^. Furthermore, the Bio-C Sealer was used due to its adequate flow and filling capacity ^21^. However, bioceramic sealers have lower bond strength to dentin compared to AH Plus ^22^. The chemical adhesion on the material/dentin interface and the greater penetration of endodontic sealers in the dentinal tubules can prevent displacement of the filling material ^18^. Thus, materials with greater adhesion to dentin can prevent the formation of voids in the root canal obturation after apicoectomy. In addition, future studies should be performed to investigate the influence of other thermoplastic obturation techniques on the adaptation of the material to the dentinal walls after apicoectomy.

Micro-CT makes it possible to quantify voids inside the filling material, using high-resolution images ^19^. However, micro-CT images may show artifacts in the presence of endodontic sealers ^19^ ^,^ ^23^. On the other hand, specific tools available in the reconstruction software can be used to optimize the acquisition parameters ^24^. In this study, tools such as “ring artifact reduction”, “beam hardening reduction” and “smoothing attenuation histogram” were used to reconstruct the images, enabling the operator to reduce artifacts and ensure reliable analysis ^23^ ^,^ ^24^. In addition, the images in the present study were acquired using a high-resolution protocol, with a voxel size of 8.74 μm, which may contribute to the reduction of the beam-hardening phenomenon ^19^ ^,^ ^23^.

Displacement of obturator material during apicectomy has an impact on success ^10^. Our results showed similar maladaptation of filling material to dentin between the tested devices. Therefore, to improve root canal sealing after apicectomy, the use of retrograde obturation technique should always be considered. The use of the ultrasonic tip presents advantages that must be considered, such as better access to the root apex, mainly in molars ^6^. In addition, the association of the operating microscope with ultrasonic tips improves visibility, increasing the precision and predictability of periradicular surgeries ^25^.

Within the limitations of this *ex-vivo* study, it can be concluded that Endo-Z bur and Bladesonic ultrasonic tip promoted increased voids at the material/dentin interface. Apicoectomies performed with Endo-Z were faster compared to Bladesonic.
